# Effect of triazine thiols on steady-state mRNA levels in iPSC-derived hepatocytes

**DOI:** 10.17912/micropub.biology.002062

**Published:** 2026-03-06

**Authors:** Carla Martinez-Morant, Jui-Tung Liu, Yu-Lin Jiang, Josef Blaszkiewicz, Stephen A. Duncan

**Affiliations:** 1 Regenerative Medicine and Cell Biology, Medical University of South Carolina, Charleston, SC, US

## Abstract

We previously reported that triazine thiols reduce apolipoprotein B (ApoB) secretion from human iPSC-derived hepatocytes (HLCs) and from humanized mice. To determine whether these compounds affected hepatocyte mRNA levels, we performed bulk RNA sequencing of HLCs treated with the triazine thiol DL-1 or with vehicle (DMSO) for 24 hours. Analyses revealed that in triazine thiol-treated cells, 145 mRNAs were reduced and 37 increased by ≥ 2-fold. &nbsp;Several mRNAs encoding cysteine-rich metallothionines were upregulated, implying that HLCs respond to treatment by mounting a protective response through metal buffering.

**Figure 1. &nbsp;Impact of triazine thiol treatment on steady-state mRNA levels in hepatocyte-like cells:**
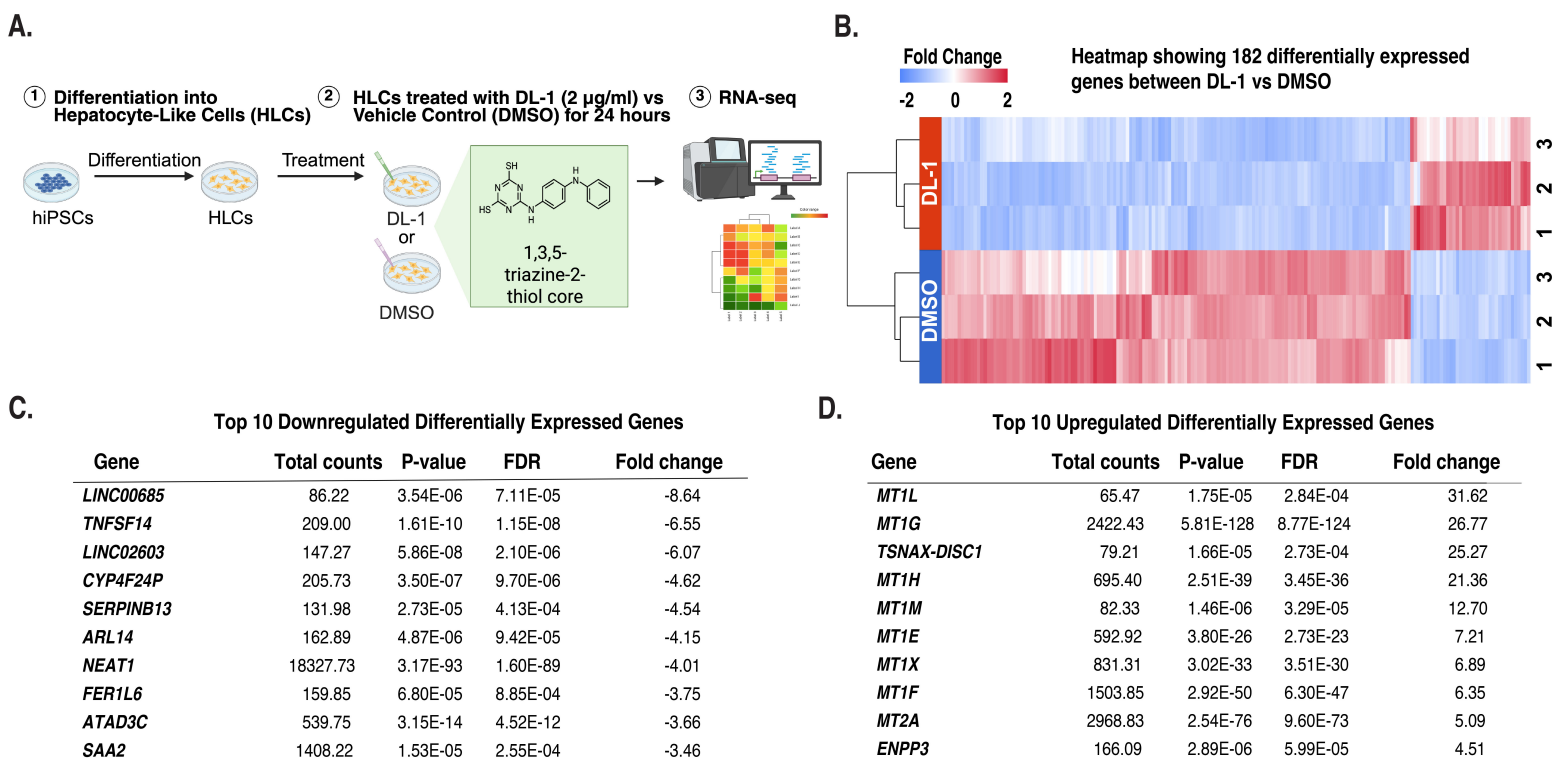
A)&nbsp;&nbsp;&nbsp; Graphical abstract of experimental design (Biorender.com). iPSCs were differentiated into hepatocyte-like cells (HLCs) for 20 days, after which they were treated for 24 hours with 2 µg/mL DL-1 or DMSO. RNA was extracted from the treated samples, and RNA sequencing was performed. B)&nbsp;&nbsp;&nbsp; Heatmap showing hierarchical cluster analyses of 182 differentially expressed genes with 37 upregulated (red) and 145 downregulated (blue); FDR ≤&nbsp;0.001, p-val ≤&nbsp;0.001, |FC| ≥ 2. C)&nbsp;&nbsp; Table showing the top 10 genes whose expression was decreased in response to triazine thiol treatment. D)&nbsp;&nbsp; Table showing the top 10 genes whose expression was increased in response to triazine thiol treatment.

## Description

The liver plays a central role in regulating plasma lipid levels by synthesizing and secreting apolipoprotein B (ApoB), which is the key protein component of low-density lipoproteins (LDL) (Whitfield et al., 2004). Excessive LDL secretion contributes to familial hypercholesterolemia (FH), which is caused by mutations in the LDL receptor (Hopkins et al., 2011). Therapeutic approaches that target ApoB synthesis or lipoprotein assembly are effective but cause excessive lipid accumulation within hepatocytes (Rader & Kastelein, 2014; Cayo et al., 2012). We previously identified DL-1, a small molecule characterized by a 1,3,5-triazine-2-thiol core (Liu et al., 2023). This molecule has therapeutic potential for FH and other dyslipidemias due to its ability to reduce ApoB secretion in induced pluripotent stem cell-derived hepatocyte-like cells (HLCs) and in humanized mice without causing lipid buildup.

We previously demonstrated that triazine thiols do not affect intracellular steady-state *APOB mRNA* or protein levels but instead affect APOB secretion (Liu et al., 2023). Although the target of the triazine thiols is unknown, the absence of an effect on *APOB* mRNA levels suggested that the triazine thiols did not directly impact transcription. Nevertheless, we felt it important to determine whether triazine thiols had a substantial effect on hepatocyte gene expression more broadly. We therefore performed bulk RNA sequencing on HLCs treated with either DL-1 at 2 µg/mL or with a DMSO vehicle control for 24 hours ( Figure 1A ). The experiment compared biological triplicates for each condition ( Figure 1B ). We selected this compound concentration because it corresponds to the EC _50_ for DL-1 (Liu et al., 2023). The effect of the compound was measured after 24 hrs, consistent with our previous analyses, which showed reduced APOB secretion at this time point (Liu et al., 2023).

Transcriptome analysis identified 182 genes whose steady-state mRNA levels were changed following DL-1 treatment (FDR ≤ 0.001, p-value ≤ 0.001, Fold change [-2,2]) ( Figure 1B ; see Extended Data File for full list of Differentially Expressed Genes (DEGs)). Of these differentially expressed genes 37 were increased and 145 were reduced. Ontology analyses revealed that genes with reduced mRNA levels exhibited diverse biological effects, with little enrichment for specific pathways or processes. The top 10 reduced genes are shown in Figure 1C . In contrast to downregulated genes, 37 genes whose steady-state mRNA levels increased were highly enriched for metallothionein genes (MT1L, MT1G, MT1H, MT1M, MT1E, MT1X, MT1F, and MT2A) (Figure 2D). This profile is characteristic of metal-responsive transcription factor 1 (MTF-1) activation, which occurs when cells’ intracellular zinc or other divalent cations are perturbed. Given DL-1’s triazine-2-thiol structure, this increase in metallothionine expression could potentially be explained by the compound’s thiol structure chelating zinc or other divalent metal cations, depleting free intracellular pools, thereby triggering an increase in metallothioneins.

## Methods

**Cell Culture and Differentiation**

Cell cultures were routinely screened for mycoplasma. Human male K3 iPSCs were generated from foreskin fibroblasts (ATCC CRL2097), and their detailed characterization, karyotyping, and short tandem repeat (STR) analyses have been previously described (Si-Tayeb, Noto, Sepac, et al., 2010). iPSCs were cultured in mTeSR medium &nbsp;supplemented with 40 ng/ml zebrafish basic fibroblast growth factor (Ludwig et al., 2006) on an E-cadherin-IgG Fc fusion protein matrix &nbsp;in 4% O2-5% CO2 incubators (Nagaoka et al., 2010).&nbsp; To initiate differentiation, K3 cells were seeded as a monolayer on Matrigel (2 mg/ml)-coated tissue culture plates for a full 24 hours prior. We induced these cells to differentiate into hepatocyte-like cells following an established protocol (Si-Tayeb, Noto, Nagaoka, et al., 2010). In the initial two days of differentiation, the cells are cultured in RPMI 1640 Medium (Invitrogen, MA, #22400105), enriched with 2% B27 Supplement without insulin (Invitrogen, MA, #A1895601), 100 ng/mL Activin A (Invitrogen, MA, #PHC9563), 20 ng/mL Fibroblast Growth Factor 2 (FGF2) (Invitrogen, MA, #PHG0023), and 10 ng/mL BMP4 (Invitrogen, MA, #PHC9533). Over the next 3 days, the cells were induced to form definitive endoderm by culturing in B27 Supplement without insulin and 100 ng/mL Activin A. Over the next 5 days, the cells were treated with B27 Supplement containing insulin, 10 ng/mL FGF2, and 20 ng/mL BMP4 to generate hepatic progenitor cells. The cells then received an additional 5 days of B27 Supplement and 20 ng/mL Hepatocyte Growth Factor (Invitrogen, MA, #PHG0321) to generate immature hepatocytes. Finally, the cells were cultured in HCM medium (Lonza, MD, #CC3198) and supplemented with 20 ng/mL Oncostatin M (Invitrogen, MA, #PHC5015) for the final 5 days of differentiation, thereby inducing hepatocyte-like cell differentiation (Mallanna & Duncan, 2013).

**DL-1 Treatment**

On day 20 of differentiation, cells were treated with DL-1 (2 µg/mL) or vehicle control (DMSO) in maturation medium for 24 hours, with three biological replicates per condition.

**RNA Extraction and Sequencing**

After treatment, cells were washed with PBS, and RNA was extracted using the RNeasy Plus Mini Kit (QIAGEN). High-throughput sequencing was performed by Beijing Genomics (Shenzhen, China), which confirmed RNA integrity and performed library preparation (Illumina).

**Bioinformatics Analysis**

RNA-seq bioinformatics analysis was performed on FASTQ files using Partek Genomics Suite version 12.3.1. STAR was used to align the reads, and the resulting alignments were annotated to the human genome (hg38). Counts per Million (CPM) were used for normalization. DEseq2 (R) was used to conduct differential expression analysis and statistical significance was reached at p-value (≤&nbsp;0.001), FC (-2,2), and FDR (≤&nbsp;0.001). Pathway Enrichment Analysis was performed on the DEGs using Enrichr against GO Biological Processes 2025 and Reactome Pathways 2024. Heatmaps were generated using Science Machine, Inc. (2026).

The RNA-seq dataset generated in this study has been deposited in GEO (GSE318437).

## Reagents

**Table:** 

Reagent / Resource	Description	Available from / Methods
K3 human iPSCs	Human male induced pluripotent stem cells derived from foreskin fibroblasts	Si-Tayeb et al., 2010
mTeSR Medium	Maintenance medium for human iPSCs	Ludwig et al., 2006 &nbsp;
Zebrafish basic fibroblast growth factor (bFGF)	Supplement for iPSC maintenance (40 ng/mL)	Ludwig et al., 2006
E-cadherin-IgG Fc fusion protein matrix	Defined matrix for feeder-free iPSC culture	Nagaoka et al., 2010
Matrigel (Geltrex)	Basement membrane matrix (2 mg/mL)	ThermoFisher Scientific, NY, #A1413302 &nbsp;
RPMI 1640 Medium	Basal medium for early differentiation	ThermoFisher Scientific, NY, #22400089 &nbsp;
B27 Supplement without insulin	Supplement for definitive endoderm induction	ThermoFisher Scientific, NY, #A1895601
B27 Supplement with insulin	Supplement for hepatic progenitor and maturation stages	ThermoFisher Scientific, NY, #17504044
Activin A	Growth factor for definitive endoderm induction (100 ng/mL)	ThermoFisher Scientific, NY, #PHC9563
Fibroblast Growth Factor 2 (FGF2)	Growth factor for hepatic specification (20 ng/mL)	ThermoFisher Scientific, NY, #PHG0023
Bone Morphogenetic Protein 4 (BMP4)	Growth factor for hepatic progenitor induction (20 ng/mL)	ThermoFisher Scientific, NY, #PHC9533
Hepatocyte Growth Factor (HGF)	Growth factor for immature hepatocyte differentiation (20 ng/mL)	Invitrogen, MA, USA, #PHG0321
Hepatocyte Culture Medium (HCM)	Maturation medium for hepatocyte-like cells	Lonza, MD; #CC3198
Oncostatin M	Cytokine for hepatocyte maturation (20 ng/mL)	Invitrogen, MA, USA, #PHC5015
DL-1	Triazine-2-thiol compound used for treatment (2 µg/mL)	Liu et. al. , 2023
Dimethyl sulfoxide (DMSO)	Vehicle control for DL-1 treatment	Sigma-Aldrich #67-68-5
RNeasy Plus Mini Kit	RNA extraction kit	QIAGEN #74136
Partek Genomics Suite v12.3.1	RNA-seq data processing and normalization	Illumina San Diego, CA 92122 USA. &nbsp;
STAR aligner	RNA-seq read alignment to human genome (hg38)	Illumina San Diego, CA 92122 USA.
DESeq2(R)	Differential gene expression analysis	Illumina San Diego, CA 92122 USA.
Enrichr	Pathway enrichment analysis (GO BP 2025, Reactome 2024)	Maayan Lab, Icahn School of Medicine, NY
Science Machine AI platform	Bioinformatic Analysis Platform	Science Machine, Inc. (2026)
RNA-seq data	This study	GEO: GSE318437

## Data Availability

Description: Excel Table of all DEGs. Resource Type: Dataset. DOI: https://doi.org/10.22002/ecahh-znn24
